# A novel G protein-coupled receptor for starfish gonadotropic hormone, relaxin-like gonad-stimulating peptide

**DOI:** 10.1371/journal.pone.0242877

**Published:** 2020-11-23

**Authors:** Masatoshi Mita, Shin Matsubara, Tomohiro Osugi, Akira Shiraishi, Azumi Wada, Honoo Satake

**Affiliations:** 1 Department of Biochemistry, Showa University School of Medicine, Tokyo, Japan; 2 Bioorganic Research Institute, Suntory Foundation for Life Sciences, Kyoto, Japan; Shanghai Ocean University, CHINA

## Abstract

Gonadotropic hormones play important regulatory roles in reproduction. Relaxin-like gonad-stimulating peptide (RGP) is a gonadotropin-like hormone in starfish. However, a receptor for RGP remains to be identified. Here, we describe the identification of an authentic receptor for RGP (RGPR) in the starfish, *Patiria pectinifera*. A binding assay using radioiodinated *P*. *pectinifera* RGP (PpeRGP) revealed that RGPR was expressed in ovarian follicle cells. A RGPR candidate was identified by homology-searching of transcriptome data of *P*. *pectinifera* follicle cells. Based on the contig sequences, a putative 947-amino acid PpeRGPR was cloned from follicle cells. Like the vertebrate relaxin family peptide receptors (RXFP 1 and 2), PpeRGPR was a G protein-coupled receptor that harbored a low-density lipoprotein-receptor class A motif and leucine-rich repeat sequences in the extracellular domain of the N-terminal region. Sf9 cells transfected with Gαq_16_-fused PpeRGPR activated calcium ion mobilization in response to PpeRGP, but not to RGP of another starfish *Asterias amurensis*, in a dose-dependent fashion. These results confirmed the species-specific reactivity of RGP and the cognate receptor. Thus, the present study provides evidence that PpeRGPR is a specific receptor for PpeRGP. To the best of our knowledge, this is the first report on the identification of a receptor for echinoderm RGP.

## Introduction

Starfish are suitable animals for studying the regulatory mechanism of oocyte maturation and ovulation, because the gonadotropic hormone and the maturation-inducing hormone (MIH) were first identified in starfish among invertebrates [1–3]. In vertebrates, gonadotropins (follicle stimulating hormone and luteinizing hormone) secreted from the pituitary gland are glycoproteins with a molecular weight of around 30 kDa. While there is no pituitary gland in starfish, gonadotropin-like activity was found in the extract of radial nerve cords in starfish over 60 years ago [4]. The active substance in starfish had been first designated as gamete-shedding substance [5,6], and subsequently gonad-stimulating substance (GSS) [7]. However, the chemical structure of the gonadotropic hormone of starfish remained unknown for decades.

In 2009, GSS was finally purified from the radial nerve cords of starfish *Patiria* (synonym, *Asterina*) *pectinifera* and its chemical structure was identified [8]. The purified GSS was a heterodimeric peptide comprising an A- and B-chain with disulfide cross-linkages; two interchains between the A- and B-chains, and an intrachain within the A-chain [8]. The A-chain also contained a cysteine motif [CCxxxCxxxxxxxxC], which is a signature sequence of the insulin/insulin-like growth factor (IGF)/relaxin superfamily. Based on its cysteine motif, starfish GSS is classified as a member of the insulin/IGF/relaxin superfamily. Furthermore, a phylogenetic tree of the insulin/IGF/relaxin superfamily suggested that GSS belongs to the relaxin-type peptide family [8]. To adequately emphasize the characteristics of this peptide hormone, starfish gonadotropic hormone has been re-designated as a relaxin-like gonad-stimulating peptide (RGP) instead of GSS [2]. In other words, starfish RGP is a peptide hormone with a chemical structure similar to that of relaxin [1–3,8], which functions in assisting pregnancy and parturition in women [9].

Synthetic RGP of *P*. *pectinifera* (PpeRGP) induced oocyte maturation and ovulation in ovarian fragments of *P*. *pectinifera* within 30 min of incubation [8]. Additionally, spawning behavior and subsequent spawning were observed after injection with synthetic PpeRGP into the males and females of *P*. *pectinifera* with fully-grown testes and ovaries, respectively [8]. Although RGP is the primary mediator of oocyte maturation and ovulation, the effect of RGP is indirect. RGP secreted by radial nerve cords induces ovarian follicle cells to produce 1-methyladenine (1-MeAde) as a maturation-inducing hormone (MIH) in starfish [10–16]. The intervals preceding the discharge of gametes after the injection of 1-MeAde are almost equal to those found after injection of RGP, indicating that 1-MeAde production in ovarian follicle cells is triggered immediately after RGP application [7,17,18].

Although 1-MeAde is a common MIH in the class Asteroidea [14], the effect of RGP on gamete shedding is partially species-specific [4,19,20]. Recently, the chemical structures of RGPs were identified in several starfish species [21–25]. Because *P*. *pectinifera*, *Patiria miniata* and *Acanthaster planci* belong to the order Valvatida in the class Asteroidea, the chemical structures of RGP in *P*. *miniata* (PmiRGP) [21] and *A*. *planci* (AplRGP) [22] are almost identical to that of PpeRGP. The degree of amino acid sequence identity in PmiRGP and AplRGP to that of PpeRGP is 98% and 80%, respectively. In contrast, the chemical structures of RGP identified from *Asterias amurensis* (AamRGP) [23], *Asterias rubens* (AruRGP) [24] and *Aphelasterias japonica* (AjaRGP) [25] of the order Forcipulatida are quite different from that of PpeRGP. The sequence identity between Forcipulatida species (AamRGP, AruRGP and AjaRGP) and PpeRGP is around 60%. These findings suggest that some species-specificity is due to interaction between RGP and its cognate receptor.

Previous studies have shown that PpeRGP binds specifically to a membrane preparation of ovarian follicle cells of *P*. *pectinifera* [26,27] and that isolated follicle cells cultured with PpeRGP showed a dose-related increase in cyclic AMP (cAMP) production, coinciding with an increase in 1-MeAde production [8,27–29]. This strongly suggests that the action of RGP is mediated through the activation of its cognate G protein-coupled receptor (GPCR), leading to the activation of adenylyl cyclase in follicle cells [8,29,30]. However, the RGP receptor (RGPR) remains to be identified in starfish. In this study, we identified the authentic receptor of PpeRGP, PpeRGPR.

## Materials and methods

### Animals

No permit is required for collecting starfish *P*. *pectinifera*, because elsewhere in Japan there are no laws either allowing or forbidding collection of adult *P*. *pectinifera*. Adults of *P*. *pectinifera* were collected from Yokosuka, Kanagawa, Japan (35°27′ N, 139°73′ E), Ushimado, Okayama, Japan (34°60′ N, 134°14′ E), and Asamushi, Aomori, Japan (40°90′ N, 140°86′ E). Animals were kept in circulating artificial seawater at 15°C and used within two months after collection.

### Reagents

AamRGP and PpeRGP were synthesized commercially (Peptide Institute Inc., Osaka, Japan). 1-MeAde was purchased from the Sigma-Aldrich Chemical Company (St. Louis, MO, USA). All other reagents were of analytical grade. The seawater was modified Van’t Hoff’s artificial seawater (ASW) adjusted to pH 8.2 with 0.02 M borate buffer [18].

### Preparation of follicle cells

Ovarian follicle cells were separated from folliculated oocytes in *P*. *pectinifera* as described previously [31]. Briefly, the folliculated oocytes were obtained from ovaries at breeding season and were treated with ASW containing 1 μM 1-MeAde for 1 h at room temperature. Follicle cells were separated from oocytes by allowing the latter to sediment by gravity. The supernatant containing the follicle cells was collected by centrifugation at 1,000 *g* for 10 min at 4°C.

### cDNA cloning and sequencing

PpeRGPR was identified by BLAST analysis against Trinity (https://github.com/trinityrnaseq)-assembled contig sequences with transcriptome sequence data of *P*. *pectinifera* follicle cells (Accession Nos. SRR8627925 and SRR8627926) using RXFP1_human (GenBank: Q9HBX9) and RXFP2_human (GenBank: Q8WXD0) as queries. To confirm the contig sequence, a cDNA encoding the PpeRGPR was cloned and sequenced.

Total RNA was extracted from *P*. *pectinifera* follicle cells using the Sepasol reagent (Nacalai Tesque, Kyoto, Japan) as the RNA extraction solution. A Poly(A)^+^ RNA fraction was obtained using Oligotex-dT30 (Nippon Gene, Tokyo, Japan). cDNA was synthesized using the Superscript III RT (Invitrogen, Waltham, MA, USA) in accordance with the manufacturer’s instructions. A cDNA of the entire coding region of the PpeRGPR transcript was amplified by PCR using ExTaq polymerase (Takara Bio Inc., Shiga, Japan) with the oligonucleotide primers (S1 Table) (custom synthesized by Fasmac, Kanagawa, Japan). All PCR products were electrophoresed in 1.5% agarose gels and stained with ethidium bromide. Agarose gel slices containing the PCR-product band were excised under UV illumination, and DNA was purified from the agarose gel using a QIAquick^®^ Gel Extraction kit (Qiagen, Valencia, CA, USA), followed by an ethanol precipitation. Amplified products were cloned into a pGEM-T^®^ easy vector (Promega, Madison, WI, USA). DNA sequence data were determined using an ABI PRISM 3130 Genetic Analyzer (Applied Biosystems, Foster City, CA, USA) with a Big Dye Terminator v3.1 Cycle Sequencing kit (Applied Biosystems).

The Expasy translate tool (http://web.expasy.org/translate/) was used to determine the protein sequence of the PpeRGPR and SignalP-5.0 (http://www.cbs.dtu.dk/services/SignalP/) was used to predict the signal peptide. ScanProsite (https://prosite.expasy.org/scanprosite/) and InterPro (http://www.ebi.ac.uk/interpro/search/sequence/) were also used to search the domains of the LDL-receptor class A (LDLa), leucine-rich repeat (LRR), and transmembrane (TM) regions in PpeRGPR.

### Construction of expression vector of Gαq_16_-fused PpeRGPR

Functional characterization of PpeRGPR was performed using Sf9 cells and baculovirus-based expression methods, as previously reported [32]. The open reading frame (ORF) of human Gαq_16_ protein (OriGene, Rockville, MD, USA), which couples with GPCRs and triggers intracellular calcium mobilization upon binding of a specific ligand [33] was subcloned into the *Xba*I site of the pFastbacI plasmid (Invitrogen, Waltham, MA, USA). PpeRGPR was then cloned into the *Not*I/*Xba*I site of the Gαq_16_-ligated pFastbacI plasmids. Transformation of competent cells with the PpeRGPR-Gαq_16_-pFastbacI plasmid and the resulting bacmid isolation was performed according to the manufacturer’s instructions for the Bac-to-Bac system (Thermo Fisher Scientific, Waltham, MA, USA).

### Detection of calcium ion mobilization

Sf9 cells (Thermo Fisher Scientific) were grown in Sf900 II (Thermo Fisher Scientific) containing 10% FBS (Sigma-Aldrich) at 28°C. PpeRGPR-Gαq_16_-recombinant baculoviruses were prepared from Sf9 cells transfected with the above bacmids using Cellfectin II (Thermo Fisher Scientific), titrated, isolated, and transiently transfected into Sf9 cells, as previously described [32]. Sf9 cells were plated at 1.2×10^5^ cells/well (on a 96-well plate) or 2.4×10^5^ cells (on a glass bottom dish) prior to transfection. Forty-eight hours after transfection, Sf9 cells were loaded for 30 min with 2.5 μM of Fluo-8 AM (AAT Bioquest, Sunnyvale, CA, USA) diluted in loading buffer [HBSS supplemented with 1.25 mM probenecid and 0.04% (wt/vol) pluronic F-127] [32]. Cell membrane expression of PpeRGPR-fused human Gαq_16_ was confirmed by immunostaining using the anti-Gαq_16_ antibody (OriGene, TA318890) in comparison with non-transfected Sf9 cells (wild type). Dose-dependent responses to PpeRGP or AamRGP were measured using a FlexStation II-automated apparatus (Molecular Devices, San Jose, CA, USA) at the excitation/emission wavelengths of 490/514 nm [32]. Calcium accumulation data were analyzed using Prism v6 (GraphPad Software, San Diego, CA, USA) to fit to a sigmoidal concentration-response curve, as previously described [32]. The dose-response curve was statistically evaluated by analysis of variance. Significant activation of PpeRPGR by PpeRGP compared with AamRGP at each concentration was also examined by Student *t* test.

### Binding experiments

Follicle cells were homogenized using a Teflon homogenizer in 25 mM Tris-HCl (pH 7.4) containing 10 mM MgCl_2_. Protein concentrations were measured using a Bio-Rad protein assay kit (Bio-Rad Laboratories, Hercules, CA, USA).

The radioiodination of PpeRGP with Na^125^I (carrier free; GE Healthcare, Buckinghamshire, UK) was carried out at room temperature in accordance with the modified lactoperoxidase method, as described previously [34,35]. The specific activity of radioiodinated PpeRGP (^125^I-PpeRGP) was estimated to be 150 μCi/μg.

The specificity of RGP binding was examined using whole homogenates of follicle cells. Preparations were adjusted to yield a protein concentration of 10 mg/ml with the homogenizing medium plus 0.1% (wt/vol) BSA, and stored at -80°C until use. The binding assay was performed using competition experiments between labeled PpeRGP and unlabeled PpeRGP or AamRGP, as described previously [34,35]. Data were obtained from four separate assays using follicle cells from ovaries of four different animals.

### 1-MeAde and cAMP assays

Ten million follicle cells were incubated for 2 h at 20°C in 1 ml of ASW in the presence of PpeRGP or AamRGP, with occasional shaking. The cell suspension was then centrifuged at 1,000 *g* for 1 min and quickly frozen in liquid nitrogen. Supernatants were analyzed for the amount of 1-MeAde released from follicle cells. The concentration of 1-MeAde was determined by a method previously described [36], using authentic 1-MeAde as the standard reference. The frozen follicle cells were analyzed for the amount of intracellular cAMP using a BIOTRAK cAMP EIA system (GE Healthcare). The mean was determined from three separate assays using three different animals and standard error of the mean (SEM).

### Searching RGPR homologs

On the basis of the *PpeRGPR* cDNA sequence, RGPR homologs were identified by searching the sequence data bases of *P*. *miniata* and *A*. *planci*. A putative *P*. *miniata* RGPR (PmiRGPR) sequence was obtained by BLAST analysis and assembly of the transcriptome sequence data for *P*. *miniata* (BioSample: SAMN03853122; SRA: SRS074426). A relaxin receptor 2-like isoform [*Acanthaster planci*] (GenBank: XM_022245101.1) showed the highest homology to PpeRGPR and was recognized as a putative AplRGPR.

## Results and discussion

### PpeRGPR was identified as a specific receptor for PpeRGP

**Fig 1:**
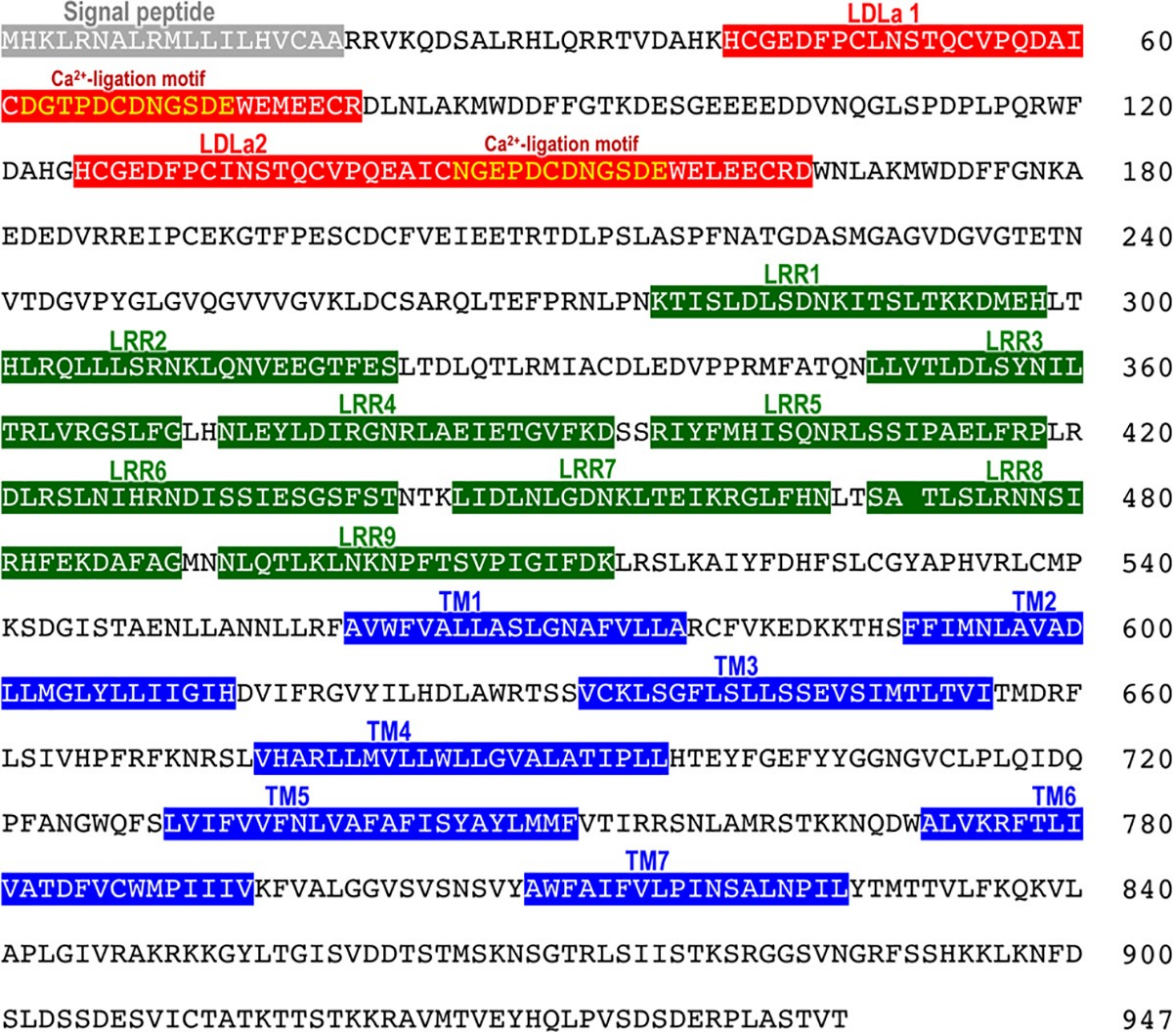
Amino acid sequence of the starfish *Patiria pectinifera* RGP receptor (PpeRGPR). The signal peptide, low-density lipoprotein-receptor class A (LDLa), leucine-rich repeat (LRR), and transmembrane domains (TM) are indicated with gray, red, green and blue backgrounds, respectively. Yellow characters indicate the Ca^2+^-ligation motifs.

Previously, a binding assay was used to demonstrate that ^125^I-PpeRGP interacts with cell membrane preparations of *P*. *pectinifera* ovarian follicle cells [34,35]. We thus obtained transcriptome sequence data for *P*. *pectinifera* follicle cells, and searched for candidate PpeRGP receptor (PpeRGPR) genes by BLAST analysis of the transcriptome using human RXFP1 and RXFP2 as queries. Two contigs (c47578 and c45649) encoding putative PpeRGPR were obtained as RXFP homologs. To confirm the contig sequences, a cDNA encoding the putative PpeRGPR was cloned and sequenced. The ORF of putative PpeRGPR consisted of 2,844 bp (S1 Fig) and encoded 947 amino acids (S2 Fig) harboring seven transmembrane (TM) domains (Fig 1). The putative PpeRGPR was found to be a GPCR (Fig 1) and to bear two low-density lipoprotein-receptor class A (LDLa) and nine leucine-rich repeat (LRR) domains in the extracellular region of the N-terminal segment (Fig 1). A calcium-binding motif [D/NxxxDxxD/NxxDE] [37] was also found in both LDLa domains (Fig 1). These structural features are reminiscent of RXFPs. These suggest that PpeRGPR belongs to subgroup δ of the rhodopsin class (Type I or Class A) of GPCRs and are further classified as subtype C LGRs [38].

**Fig 2:**
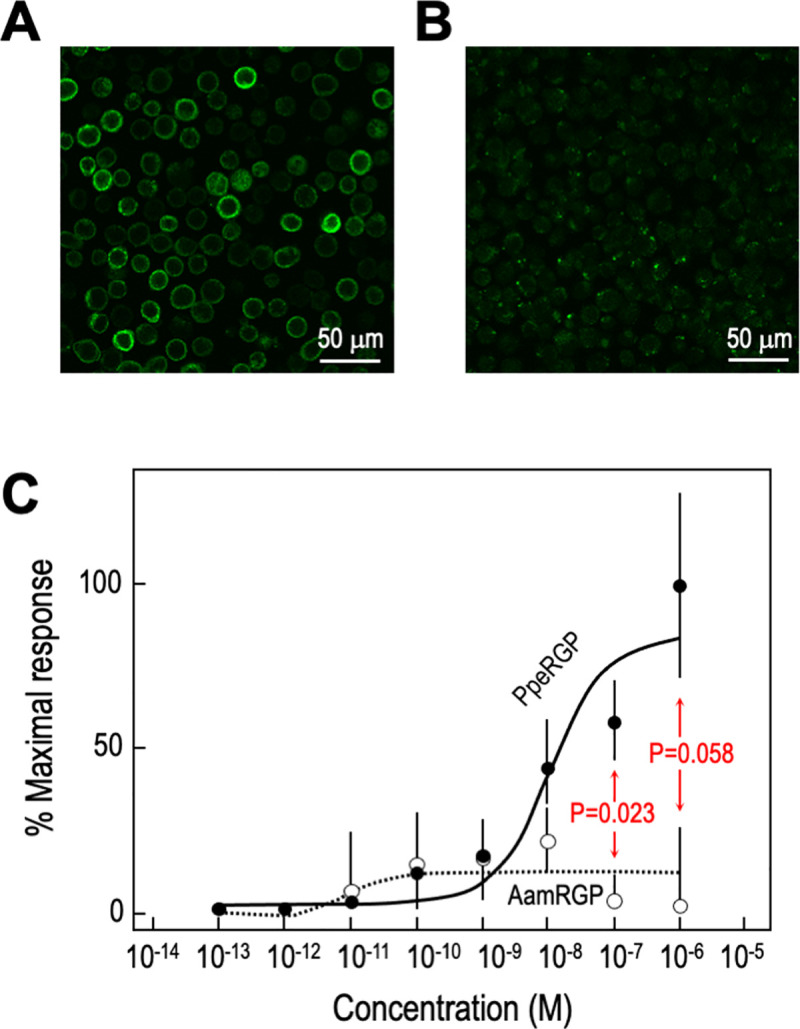
Intracellular calcium mobilization in Sf9 cells. (A, B) Sf9 cells were transfected with Gαq_16_- fused PpeRGPR-recombinant baculovirus (A) and non-transfected Sf9 cells were used as wild type controls (B). Expression of PpeRGPR in Sf9 cells was examined by immunostaining using an anti-Gαq_16_ antibody. Immunoreactivity was observed using a fluorescence microscope after incubation with an Alexa Fluor 488-conjugated secondary antibody. Specific PpeRGPR immunoreactivity was localized at the cell membrane (A), whereas no signal was observed in the control cells (B). Scale bars represent 50 μm. (C) Calcium mobilization dose-response curves for PpeRGP and AamRGP in Sf9 cells transfected with Gαq_16_- fused PpeRGPR-recombinant baculoviruses. The kinetics of real-time Fluo-8 fluorescence was observed at excitation/emission wavelengths of 490/514 nm in Sf9 cells exposed to various concentrations of PpeRGP (○) or AamRGP (●). PpeRGPR was specifically stimulated by PpeRGP in a dose-dependent fashion. Symbols and bars represent the mean ± standard error of the mean from three to seven independent samples. Each dose-response curve was statistically evaluated by analysis of variance. The difference between PpeRGP and AamRGP was also examined by Student *t* test.

To examine whether the putative PpeRGPR acts as a receptor for PpeRGP, the Gαq_16_- fused PpeRGPR gene was transfected into Sf9 cells. Immunostaining demonstrated localization of ectopic PpeRGPR at the cell membrane (Fig 2A), which was not observed in wild type cells (Fig 2B). PpeRGP significantly increased calcium signaling at concentrations above 1 nM in a dose-dependent manner (Fig 2C, EC_50_ = 11.1 nM, P = 3.23E-05). In contrast, AamRGP exhibited no effect on calcium signaling in Sf9 cells (Fig 2C, P = 0.89). Although the activation between PpeRGP and AamRGP at 1 μM was not significant (P = 0.058), that at lower concentration (100 nM) was statistically significant (P = 0.023). These results indicated that PpeRGPR specifically binds to PpeRGP and upregulates intracellular Ca^2+^ levels. Thus, PpeRGPR is an authentic receptor for endogenous PpeRGP and exhibits species-specific interactions with RGP.

### PpeRGP binds to PpeRGPR in follicle cells

**Fig 3:**
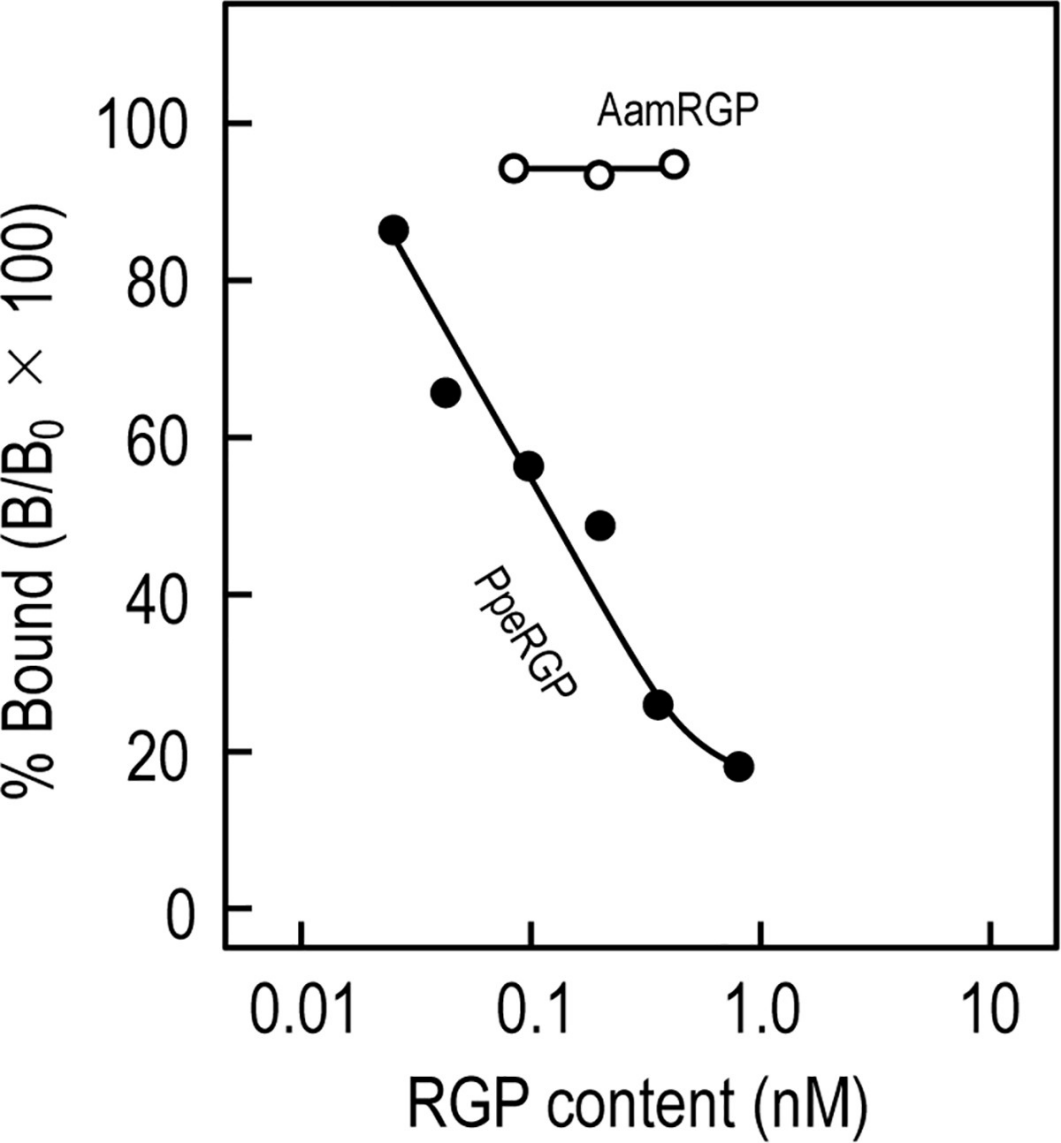
Competitive inhibition curves of ^125^I-PpeRGP binding to *Patiria pectinifera* ovarian follicle cells in the presence of unlabeled PpeRGP or AamRGP. *P*. *pectinifera* ovarian follicle cell homogenates (25 μg of protein) were incubated for 2 h at 20°C in assay buffer containing ^125^I-PpeRGP (about 100,000 cpm; 2.087 pg/100 ml; total volume 0.5 ml, final concentration 1.1 pM) in the presence of serially diluted unlabeled PpeRGP (●) or AamRGP (○). Specific binding was obtained by subtracting nonspecific binding from total binding. Data were obtained from four separate assays using follicle cells from ovaries of four different animals.

Since cDNA encoding the PpeRGPR was cloned from *P*. *pectinifera* follicle cells (Fig 1), PpeRGPR should be expressed on follicle cell surface. To elucidate if the species-specificity of PpeRGPR derives from signaling pathway or binding mechanism, a competitive binding assay between ^125^I-iodinated PpeRGP (^125^I-PpeRGP) and non-iodinated PpeRGP or AamRGP was carried out using *P*. *pectinifera* follicle cells. ^125^I-PpeRGP binding to follicle cells could be decreased in a dose-dependent manner in the presence of un-labeled PpeRGP (Fig 3). This result is in accordance with previous studies [26,27] that report PpeRGP binding to *P*. *pectinifera* follicle cells, and reveals that PpeRGPR is present in ovarian follicle cells. Moreover, Scatchard plot analysis [39] indicated that the dissociation constant (*K*d) for PpeRGP was approximately 0.67 nM. This *K*d value is highly consistent with previous data indicating that the median effective concentration of *in vitro* spawning is approximately 1–2 nM of PpeRGP [8]. In contrast, AamRGP had no effect on ^125^I-PpeRGP binding (Fig 3), suggesting that AamRGP is unable to bind to PpeRGPR in follicle cells. Furthermore, this emphasizes that PpeRGPR specifically binds to PpeRGP.

**Fig 4:**
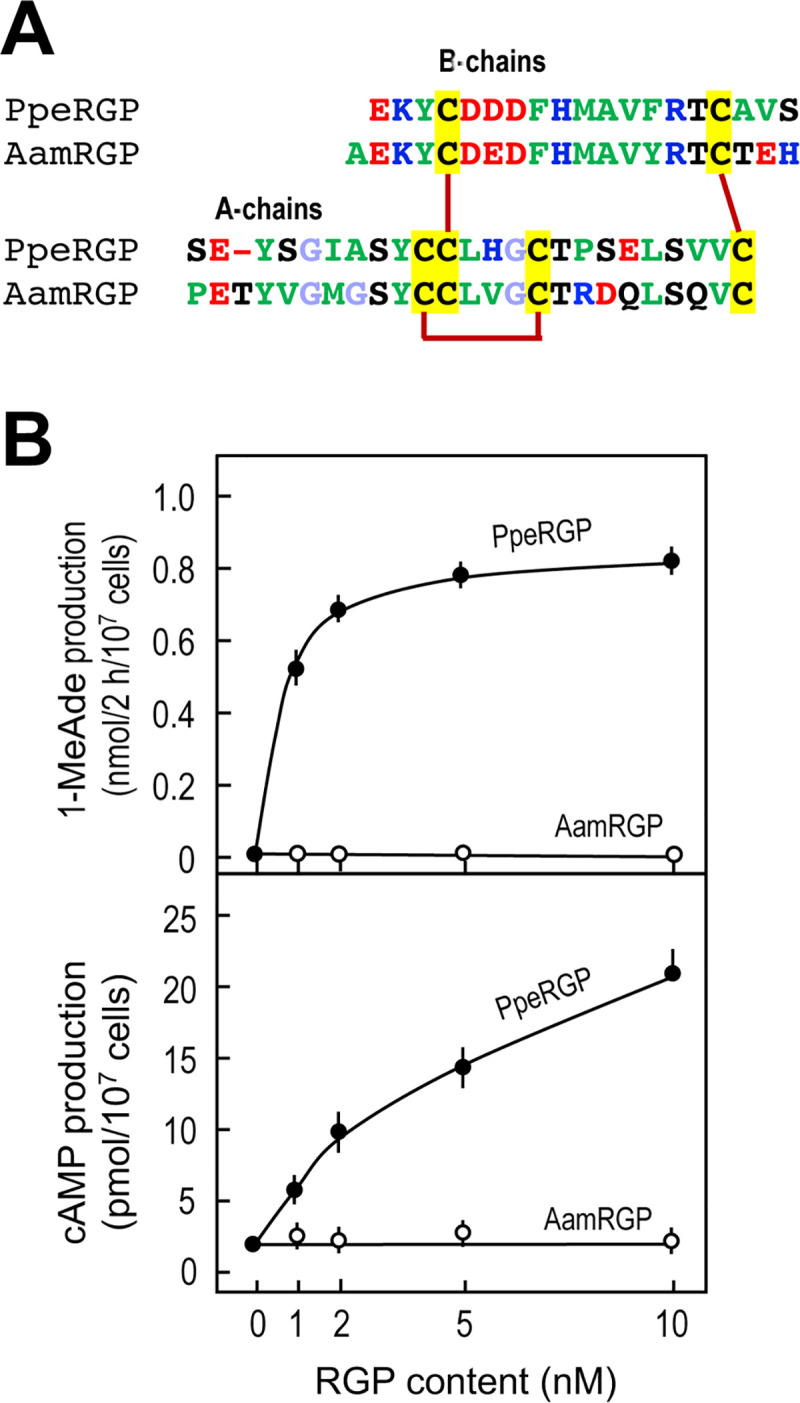
Effect of RGP on 1-MeAde and cAMP production in *Patiria pectinifera* ovarian follicle cells. (A) Amino acid alignments of A- and B-chains in *P*. *pectinifera* (PpeRGP) and *Asterias amurensis* (AamRGP). To illustrate the conserved features, the amino acid types are color coded according to their properties, with basic residues in blue (Arg, Lys and His), acidic residues in red (Glu and Asp), hydrophobic residues in green (Ala, Val, Ile, Phe, Trp, Tyr, Pro and Met), hydrophilic in black (Ser, Thr, Asn and Gln) and glycine in light blue. The cysteine residues are highlighted in yellow and disulfide bonds are shown with solid dark red lines. (B) Effect of PpeRGP and AamRGP on 1-MeAde and cAMP production in follicle cells. Follicle cells were incubated in ASW in the presence of either PpeRGP (●) or AamRGP (○) for 2 h at 20°C. The amount of 1-MeAde released into the medium was estimated using a biological assay with an analytical curve generated with authentic 1-MeAde. Intracellular cAMP content was determined by EIA. Symbols and bars represent the mean of three independent samples and standard error of the mean (SEM), respectively.

It has been demonstrated in mammals that the B-chain of relaxin and related peptides plays an important role in binding to the receptor [40–42]. Despite its similarity with the relaxin superfamily, the RGP sequence does not possess the vertebrate ‘relaxin-specific receptor-binding cassette’ (RxxxRxxI/V), a distinct and well-conserved feature of the relaxin group B-chains identified to date [43]. A comparison of the amino acid sequences of the middle region of the B-chains indicates that residues of the ‘receptor-binding cassette’ correspond to D^B6^, M^B10^ and F^B13^ for PpeRGP and E^B7^, M^B11^, and Y^B14^ for AamRGP, respectively [44] (Fig 4A). Although D/ExxxMxxF/Y may be involved in binding to its cognate receptors as similar to vertebrates, our binding assay suggested that more other amino acid residues are involved in species specificity.

Similar species-specificity was observed with biological assays using *P*. *pectinifera* ovarian follicle cells. In contrast to PpeRGP, AamRGP had no effects on binding to *P*. *pectinifera* follicle cells (Fig 3), as well as 1-MeAde and cAMP production (Fig 4B) or calcium signals in PpeRGPR-expressing Sf9 cells (Fig 2C). Previous studies have shown that a chimeric RGP derivative composed of the A-chain of AamRGP and the B-chain of PpeRGP fails to induce spawning in *P*. *pectinifera* ovaries [45]. Based on 3D structural models, P^A17^ of PpeRGP and R^A18^ of AamRGP are located near their respective B-chains [44]. Because the side chain of the arginine is positively charged and is larger than the side chain of the proline, it is likely that R^A18^ of AamRGP impairs binding to PpeRGPR. These findings are in good agreement with the results that AamRGP can neither bind to PpeRGPR expressed in Sf9 cells (Fig 2C) or follicle cell plasma membranes (Fig 3), nor induce 1-MeAde and cAMP production in *P*. *pectinifera* follicle cells (Fig 4B).

### RGPR homologs

Because *P*. *miniata* and *A*. *planci* belong to the same order (Valvatida) as *P*. *pectinifera* and their transcriptome sequence data were available for BLAST analysis, putative PmiRGPR and AplRGPR were searched as homologs of PpeRGPR. The DNA sequence encoding putative PmiRGPR was composed of 2,844 bp (S1 Fig) with an ORF encoding a protein of 947 amino acids (S2 Fig). The DNA sequence registered as a relaxin receptor 2-like isoform [*Acanthaster planci*] (GenBank: XM_022245101.1) was recognized to encode RGPR (AplRGPR). The coding DNA sequences of putative AplRGPR consisted of 2,817 bp (S1 Fig) and encoded 938 amino acid (S2 Fig). The coding DNA sequences of putative PmiRGPR and AplRGPR were 98% and 80% identical to that of PpeRGPR, respectively (S1 Fig). Although putative PmiRGPR and AplRGPR displayed 98% and 80% amino acid sequence identity to PpeRGPR, they shared chemical features of many amino acids with PpeRGPR (S2 Fig). On the other hand, an RGPR homolog was not found in the transcriptome sequence data of *A*. *amurensis* of the order Forcipulatida (BioSample: SAMN0316205; SRA: SRR1642063).

Four types of RXFP receptors, indicating RXFP1 [38], RXFP2 [46], RXFP3 [47] and RXFP4 [48] have been identified in vertebrates. RXFP1/2 have long extracellular domains including LDLa and LRR motifs on the N-terminal segment, whereas extracellular domains of RXFP3/4 are shorter and lack LDLa and LRR domains [40–42]. Signal transduction by RXFP1 or RXFP2 results in the activation of adenylyl cyclase followed by an increase in cAMP accumulation [41,42]. Because the action of PpeRGP is mediated through the activation of its receptor, G protein and adenylyl cyclase, leading to cAMP accumulation in follicle cells [28–30], starfish RGPR is closer to RXFP1/2 than RXFP3/4. Previous studies have shown that the orthologue genes of RXFP1/2 are present in the chordate ancestor Amphioxus [49,50]. Despite the extensive conservation of the RXFP1/2 genes in protostomes and non-chordate deuterostomes, RXFP3/4 type receptors have been found only in the genome of the ascidian *Ciona intestinalis*, but not in other invertebrate deuterostomes [50]. These findings support the view that RXFP3/4 homologs might not have been acquired in starfish.

The gonadotropic hormone in starfish is a relaxin-like peptide, RGP. This study also reveals that RGPR is a homologue of the RXFP. RGPR is close to vertebrate RXFP1/2 type receptors in having an LDLa module at the N-terminus. Because echinoderms belong to the deuterostome superphylum, starfish RGPR is thought to be derived from an ancestral protein of RXFP. Thus, identification of RGPR exhibiting distinct reproductive functions in starfish will open new avenues into the study of the evolution of relaxin-like peptides and their receptors throughout the animal kingdom with reference to their highly diversified function.

## Conclusions

The present study has elucidated the primary sequence and species-specific ligand interaction of PpeRGPR, the receptor protein for gonadotropin-like peptide (RGP) in the starfish *P*. *pectinifera*. PpeRGPR belongs to the superfamily of rhodopsin-like GPCRs. PpeRGPR harbors a long extracellular domain including LDLa and LRR motifs on the N-terminal segment, which is more structurally similar to vertebrate RXFP1/RXFP2 than RXFP3/RXFP4. Consequently, starfish RGP and RGPR might have originated from common ancestors with vertebrate relaxin family peptides and their receptors, followed by intraphyletic evolution and diversification.

## Supporting information

S1 Fig Alignment of coding DNA sequences of the *Patiria pectinifera* RGP receptor (PpeRGPR) and putative RGP receptors of *Patiria miniata* (putative PmiRGPR) and *Acanthaster planci* (putative AplRGPR). Conserved nucleotides are highlighted in green.
(TIF)

S2 Fig Alignment of amino acid sequences of the *Patiria pectinifera* RGP receptor (PpeRGPR) and putative RGP receptors of *Patiria miniata* (putative PmiRGPR) and *Acanthaster planci* (putative AplRGPR). To illustrate the conserved features, the amino acid types are color coded according to their properties, with basic residues in blue (Arg, Lys and His), acidic residues in red (Glu and Asp), hydrophobic residues in green (Ala, Val, Ile, Phe, Trp, Tyr, Pro and Met), hydrophilic in black (Ser, Thr, Asn and Gln), and glycine in light blue. The cysteine residues are highlighted in yellow.
(TIF)

S1 Table Oligonucleotide primers for cDNA cloning in PpeRGPR. (XLSX)
