# Supplementary figures and images for: A novel G protein-coupled receptor for starfish gonadotropic hormone, relaxin-like gonad-stimulating peptide
Source: PLoS One. 2020 Nov 23;15(11):e0242877. doi: 10.1371/journal.pone.0242877 (PMC7682835; doi:10.1371/journal.pone.0242877)

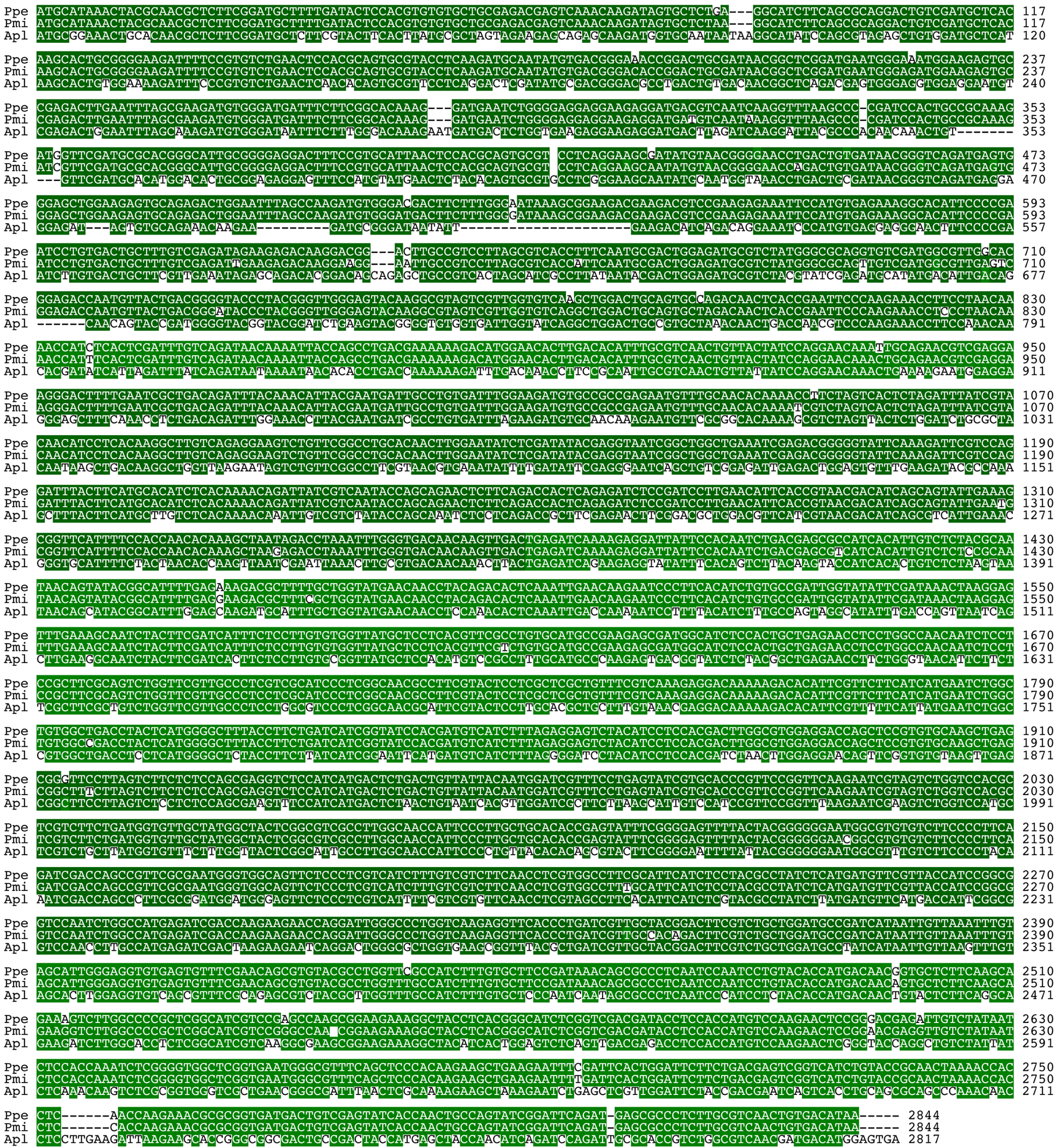

Supplement: S1 Fig — Conserved nucleotides are highlighted in green. (TIF) [file pone.0242877.s001.tif]

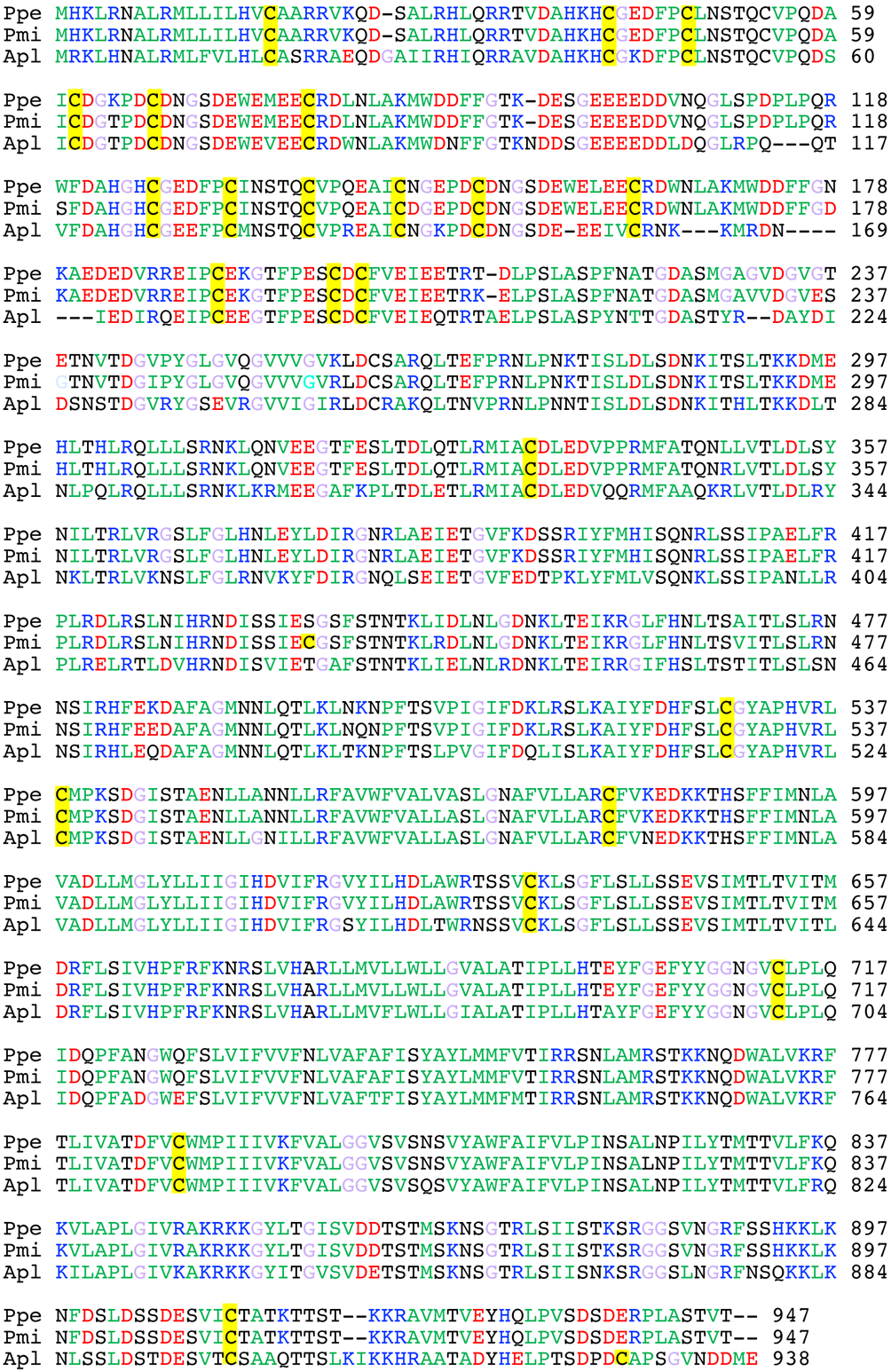

Supplement: S2 Fig — To illustrate the conserved features, the amino acid types are color coded according to their properties, with basic residues in blue (Arg, Lys and His), acidic residues in red (Glu and Asp), hydrophobic residues in green (Ala, Val, Ile, Phe, Trp, Tyr, Pro and Met), hydrophilic in black (Ser, Thr, Asn and Gln), and glycine in light blue. The cysteine residues are highlighted in yellow. (TIF) [file pone.0242877.s002.tif]
